# Photosystem II Functionality in Barley Responds Dynamically to Changes in Leaf Manganese Status

**DOI:** 10.3389/fpls.2016.01772

**Published:** 2016-11-25

**Authors:** Sidsel B. Schmidt, Marta Powikrowska, Ken S. Krogholm, Bianca Naumann-Busch, Jan K. Schjoerring, Søren Husted, Poul E. Jensen, Pai R. Pedas

**Affiliations:** Department of Plant and Environmental Sciences and Copenhagen Plant Science Centre, Faculty of Science, University of CopenhagenFrederiksberg, Denmark

**Keywords:** manganese, deficiency, barley, photosynthesis, fluorescence, photoinhibition, non-photochemical quenching

## Abstract

A catalytic manganese (Mn) cluster is required for the oxidation of water in the oxygen-evolving complex (OEC) of photosystem II (PSII) in plants. Despite this essential role of Mn in generating the electrons driving photosynthesis, limited information is available on how Mn deficiency affects PSII functionality. We have here used parameters derived from measurements of fluorescence induction kinetics (OJIP transients), non-photochemical quenching (NPQ) and PSII subunit composition to investigate how latent Mn deficiency changes the photochemistry in two barley genotypes differing in Mn efficiency. Mn deficiency caused dramatic reductions in the quantum yield of PSII and led to the appearance of two new inflection points, the K step and the D dip, in the OJIP fluorescence transients, indicating severe damage to the OEC. In addition, Mn deficiency decreased the ability to induce NPQ in the light, rendering the plants incapable of dissipating excess energy in a controlled way. Thus, the Mn deficient plants became severely affected in their ability to recover from high light-induced photoinhibition, especially under strong Mn deficiency. Interestingly, the Mn-efficient genotype was able to maintain a higher NPQ than the Mn-inefficient genotype when exposed to mild Mn deficiency. However, during severe Mn deficiency, there were no differences between the two genotypes, suggesting a general loss of the ability to disassemble and repair PSII. The pronounced defects of PSII activity were supported by a dramatic decrease in the abundance of the OEC protein subunits, PsbP and PsbQ in response to Mn deficiency for both genotypes. We conclude that regulation of photosynthetic performance by means of maintaining and inducing NPQ mechanisms contribute to genotypic differences in the Mn efficiency of barley genotypes growing under conditions with mild Mn deficiency.

## Introduction

Deficiency of essential plant nutrients is a significant problem for plant production throughout the world influencing not only crop yields but also crop quality. One of the major unsolved nutritional problems in agricultural plant production is manganese (Mn) deficiency causing substantial yield reductions and during severe winters even causing complete loss of crops (Schmidt et al., 2013; Stoltz and Wallenhammar, 2014). Even so, the actual magnitude of Mn deficiency is unknown, owing to the latency of the deficiency symptoms masking the occurrence of the problem.

Barley is particularly prone to Mn deficiency problems. The deficiency is traditionally corrected by repeatedly foliar Mn applications, often without knowing the exact Mn requirement of the plants. This method is, however, both time-consuming and inefficient, since the rate of Mn remobilisation in plants is extremely low (Loneragan, 1988). An alternative to foliar Mn applications is to select plant species or genotypes with an enhanced tolerance to growth in soils with limiting plant available Mn, a phenomenon commonly referred to as Mn efficiency (Graham, 1988; Ascher-Ellis et al., 2001; Hebbern et al., 2005). The exact mechanisms behind Mn efficiency are, however, still not fully understood (Leplat et al., 2016; Schmidt et al., 2016), but have so far been related to differential root acquisition of Mn, e.g., by the exudation of organic acid anions and phytases into the rhizosphere (Rengel and Marschner, 2005; George et al., 2014), increased Mn uptake capacity by expression of high affinity Mn transporters in the root (Pedas et al., 2005, 2008), and internal utilization of Mn in the plant and processes linked to the stability and efficiency of the photosynthetic apparatus (Husted et al., 2009; Schmidt et al., 2015).

A number of enzymatic processes in plants are activated by Mn but only a few enzymes specifically require Mn as the active site (Marschner, 2012). These include the Mn superoxide dismutase (Mn-SOD), an enzyme responsible for scavenging of reactive oxygen species in peroxisomes and mitochondria (Flohé and Ötting, 1984; Scandalios, 1993; Alscher et al., 2002), oxalate oxidase and, not least, the Mn cluster of photosystem II (PSII; Ono et al., 1992; Umena et al., 2011). The latter is situated in the oxygen evolving complex (OEC) of PSII and is responsible for the photolytic oxidation of water, releasing molecular oxygen, protons and electrons that initiate the photosynthetic electron flow. Hence, Mn deficiency leads to a rapid reduction in oxygen evolution and photosynthetic activity as the OEC is the primary target when plants are exposed to Mn deficiency (Nable et al., 1984; Kriedemann et al., 1985). In higher plants, three extrinsic proteins, PsbO, PsbP, and PsbQ shield and protect the Mn cluster by forming a triangular crown-like structure encircling the luminal binding domains of the D1 and CP43 core proteins that anchor the Mn cluster to PSII (Wei et al., 2016). The three extrinsic proteins serve to optimize oxygen evolution and efficiency of PSII, and the abundance of PsbP and PsbQ, but not PsbO, is significantly reduced under Mn deficiency (de Bang et al., 2015). In PsbP-deficient tobacco, the Mn cluster is remarkably unstable and PSII without PsbP is hypersensitive to light (Ifuku et al., 2005). In general, PSII is more affected by Mn deficiency than PSI, reflecting the significant reduction in the abundance of the extrinsic OEC proteins and the D1 core protein (Husted et al., 2009; de Bang et al., 2015).

The absorption of light by chlorophyll (Chl) and other pigments is essential to drive the photochemical reactions of photosynthesis. To understand photosynthetic reactions Chl *a* fluorescence analysis is commonly used as it reflects the electron transport processes of photosynthesis (Baker, 2008). When illuminating a leaf, kept in darkness for a short amount of time, Chl *a* fluorescence induction shows as characteristic OJIP transients rising from the minimal fluorescence (O) to fluorescence maxima (P) through the two intermediate steps called J and I. The OJIP transients can be directly correlated with processes taking place during the successive reduction of electron acceptors of the complete photosynthetic electron transport chain (Stirbet and Govindjee, 2011). By extracting values of the minimum (*F*_0_) and maximum (*F*_m_) fluorescence parameters, the PSII efficiency can be calculated as the ratio of *F*_v_ (*F*_m_-*F*_0_) to *F*_m_. This *F*_v_*/F*_m_ parameter is a very sensitive indicator of photosynthetic performance and has been proven a powerful tool to accurately diagnose even latent Mn deficiency (Schmidt et al., 2013; Leplat et al., 2016).

Plants need to balance the absorption of light in order to minimize photodamage to PSII. The photoprotective mechanisms include dissipation of absorbed excess light energy through processes such as non-photochemical quenching (NPQ). In addition, the D1 core protein of PSII may be sacrificed to protect the rest of PSII against oxidative damage under excess light. Damaged D1 proteins are continuously replaced by newly synthesized copies, providing an efficient repair cycle of PSII which allows plants to survive light stress (Aro et al., 1993; Tikkanen et al., 2014; Järvi et al., 2015). Plants exposed to additional environmental stress conditions such as Mn deficiency are more prone to photodamage owing to a dysfunctional Mn cluster of PSII (Krieger et al., 1998).

Genotypic differences with respect to photosynthetic performance under Mn deficiency have been reported based on observations that a Mn-inefficient genotype was unable to fine-tune photosynthesis by performing state transitions while a Mn-efficient genotype maintained this ability (Husted et al., 2009). The Mn-efficient genotype also incorporated more Mn per unit PSII under control and mild Mn deficiency conditions than the Mn-inefficient genotype, despite having lower or similar leaf Mn concentrations (Schmidt et al., 2015).

The aim of the present work was to characterize the dynamic changes in photochemistry induced by Mn deficiency with particular focus on damage to the OEC. We hypothesized that genotypic differences in Mn-efficiency were associated with short-term regulatory mechanisms affecting NPQ and ability to cope with photoinhibitory stress during incipient Mn deficiency. To test this hypothesis, we measured fluorescence induction kinetics, NPQ and PSII subunit composition in two contrasting barley genotypes growing at different degrees of Mn deficiency. By this experimental strategy, we attained a set of parameters which allowed the functional impact on PSII of various stages of Mn deficiency to be analyzed, thereby expanding the current knowledge on Mn deficiency and efficiency in barley (Husted et al., 2009; Schmidt et al., 2013).

## Materials and Methods

### Cultivation of Plants

Seeds of two barley genotypes with differential Mn efficiency, viz. the Mn-inefficient cv. Antonia and the Mn-efficient cv. Vanessa (Hebbern et al., 2005) were germinated for 5 days in vermiculite and subsequently transplanted to light-impermeable 4-L black cultivation units with four plants per unit. A chelate-buffered nutrient solution was prepared in double ionized water as specified in Pedas et al. (2005). The nutrient solution was renewed once a week and pH was adjusted every 3rd day to 6.0 ± 0.5 with 1 M HCl or 1 M NaOH. For the first 3 weeks, all plants received a Mn^2+^ concentration of 100 nM in the nutrient solution per week whereupon Mn-replete (control) plants received a Mn^2+^ concentration of 500 nM per week. In the remaining cultivation units, Mn was removed completely for different lengths of period (3–4 days between each Mn deficiency level) to obtain Mn deficiency levels at three different severities. The progression of Mn deficiency was continuously followed using Chlorophyll (Chl) *a* fluorescence as a tool to monitor the Mn status of the plants ensuring the establishment of three Mn deficiency levels of increasing intensity that were identical for both genotypes. The three Mn deficiency levels are hereafter designated as mild, moderate, and strong Mn deficiency. A re-supply treatment was performed by a single addition of 1,000 nM Mn^2+^ to plants exposed to strong Mn deficiency. At 8–10 days after Mn re-supply, Chl *a* fluorescence measurements demonstrated values comparable to control plants. All plants were grown in a controlled growth chamber with a 250–280 μmol m^-2^ s^-1^ photon flux density, 75–80% air humidity, and a 20°C/15°C (16 h/8 h) day/night temperature and light regime.

### Chlorophyll *a* Fluorescence Measurements

Chlorophyll *a* fluorescence induction kinetics was measured on the youngest fully emerged leaves by use of a hand-held portable fluorescence detector (Handy Plant Efficiency Analyser; Hansatech Instruments, King’s Lynn, UK) to determine the maximum quantum yield of PSII (*F*_v_*/F*_m_). The leaves were dark-adapted for 30 min using leaf clips before measurement. Fluorescence measurements were recorded by illumination for 1 s with 1,500 μmol photons m^-2^ s^-1^ at a wavelength of 650 nm.

### Fluorescence Quenching Analyses

Non-photochemical quenching (NPQ), effective quantum yield of PSII (Φ_PSII_), and PSII excitation pressure (1-qP) were measured on the youngest fully emerged leaves using a pulse amplitude modulation fluorometer (DUAL-PAM-100, Walz, Germany). Leaves were dark-adapted for minimum 30 min before measurements. Data were analyzed using the software version, DUAL-PAM version 1.11. Fluorescence were measured on leaves either in continued growth light being 300 μmol photons m^-2^ s^-1^ (PAR) or increasing PAR levels from 0–1,976 μmol photons m^-2^ s^-1^ PAR with 1 min exposure per light level.

### Element Concentrations in Leaves

The youngest fully emerged leaves were freeze dried (Christ Alpha 2–4; Martin Christ GmbH, Osterode, Germany). Homogenized leaf material was digested with ultra-pure acids (HNO_3_ and H_2_O_2_) as previously described (Pedas et al., 2005; Hansen et al., 2009). Subsequently, the element concentrations were analyzed by ICP-OES (Optima 5300 DV, PerkinElmer, USA). The data quality was evaluated by including certified reference material (apple leaf, NIST1515, National Institute of Standards and Technology, Gaithersburg, MD, USA) in each analytical run. Data was accepted if the accuracy was above 90% of certified reference values.

### Isolation of Thylakoid Membranes

Isolation of thylakoid membranes were performed under dim green light at 5°C. Leaves were disrupted in a razor-blade blender in ice-cold homogenisation buffer [0.2 M sucrose, 10 mM NaCl, 5 mM MgCl_2_, 20 mM Tricine (pH 7.9), 10 mM ascorbate and 10 mM NaF, 1mM Na-orthovanadate, and 1 tablet of protease inhibitor (Complete protease inhibitor, Roche) per 100 mL buffer]. The extract suspension was filtered through two layers of Miracloth and centrifuged at 6,000 × *g* for 10 min at 4°C. The supernatant was decanted and the pellet was re-suspended in Tricine buffer [5 mM Tricine (pH 7.9), 10 mM NaF, 1mM Na-orthovanadate, and 1 tablet of protease inhibitor per 100 mL buffer] followed by 10 min centrifugation at 11,200 × *g*, at 4°C. The thylakoid pellet was re-suspended in storage buffer [0.2 M sucrose, 10 mM NaCl, 5 mM MgCl_2_, 20 mM Tricine, 10 mM NaF, 20% glycerol, 1 mM Na-orthovanadate, and 1 tablet of protease inhibitor per 100 mL buffer] and stored at -80°C until further analysis.

### Western Blotting

Thylakoid samples (equivalent of 2 μg of total protein) were subjected to SDS-PAGE as described in Powikrowska et al. (2014) or by using the following conditions: 12% Criterion TGX Stain-Free precast gels (Bio-Rad, USA) and Tris/Glycine/SDS running buffer (Bio-Rad, USA) at 250 V constant for 32 min. The separated proteins were transferred to a 0.2 μm PVDF membrane using *Trans*-Blot Turbo transfer system (Bio-Rad, USA) according to the protocol of the manufacturer. Subsequently, the membrane was blocked for 1 h in 5% (w/v) skimmed milk in PBS-T buffer and incubated overnight in primary antibody. All primary antibodies were obtained from Agrisera, AB, Sweden. CF1-ATPase was used as a loading control. The blot was washed three times 5 min in PBS-T buffer and incubated with a secondary swine anti-rabbit horseradish HRP-conjugated antibody (Pierce, USA) at a 1:5,000 dilution in PBS-T for 1 h. Again the blot was washed three times 5 min in PBS-T buffer and the secondary antibody was detected using Clarity Western ECL chemiluminescent substrate (Bio-Rad, USA) and developed with a ChemiDoc Touch Imaging System (Bio-Rad, USA). Quantification of the protein bands was performed using Image Lab software (version 5.2.1, Bio-Rad laboratories, USA). Data was normalized to the CF1-ATPase signal, and the effects of the Mn treatments were interpreted by calculating the relative ratio between treatment and control.

### Data Analysis

Statistical analysis was undertaken using SAS (SAS Institute; version 9.3.) or R (R version 3.2.1) for variance analysis for comparison of mean values of *F*_v_*/F*_m_ values, leaf Mn concentrations, and protein abundances between the treatments. The genotypic effect of Mn deficiency on *F*_v_*/F*_m_ and leaf Mn concentrations were analyzed with two-way ANOVA (**Table 1**) and the effect of Mn deficiency on protein abundance was analyzed with one-way ANOVA (**Figure 5**) for each genotype. Both analyses were followed by LSD test and *P*-values < 0.05 were considered significant. Mean values (*X*) are listed with the associated SE values (X ±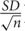).

**Table 1:** Maximum quantum yield of photosystem II (*F*_v_*/F*_m_) and corresponding leaf Mn concentrations (μg g^-1^ dry weight) in the youngest fully emerged leaves of the Mn-inefficient genotype Antonia and the Mn-efficient genotype Vanessa growing under control, Mn-deficient or Mn-resupplied conditions.

	*F*_v_*/F*_m_		Leaf Mn concentration (μg g^-1^ DW)	
	Antonia	Vanessa	Antonia	Vanessa
Control	0.83 ± 0.01^a^	0.83 ± 0.01^a^	16.8 ± 0.9^b^	20.0 ± 1.6^a^
Mild Mn deficiency	0.70 ± 0.01^b^	0.68 ± 0.01^b^	8.8 ± 0.2^c^	9.3 ± 0.2^c^
Moderate Mn deficiency	0.59 ± 0.01^c^	0.59 ± 0.01^c^	6.2 ± 0.2^cd^	6.7 ± 0.2^cd^
Strong Mn deficiency	0.50 ± 0.01^d^	0.50 ± 0.01^d^	3.8 ± 0.3^d^	5.3 ± 0.1^d^
Re-supply	0.82 ± 0.01^a^	0.82 ± 0.01^a^	18.8 ± 0.2^ab^	17.5 ± 0.1^ab^

*Results are means ± SE (*n* = 3-6 for Mn concentrations, *n* = 12-24 for *F*_v_*/F*_m_). Values followed by the same letter are not significantly different (*P* ≥ 0.05).*

## Results

### Establishment of Plants with Controlled Mn Deficiency

Two barley genotypes with contrasting Mn efficiency were grown at different levels of Mn deficiency, designated as control, mild, moderate, or strong Mn deficiency. Chl *a* fluorescence was continuously measured to monitor the progression of Mn deficiency and revealed the induction of Mn deficiency below a critical threshold limit of about 15 μg Mn g^-1^ dry weight (DW) in barley leaves (**Table 1**) (Reuter et al., 1997), although no visual leaf Mn deficiency symptoms could be observed in any of the treatments. However, it should be noted that prevailing Mn deficiency eventually will bring about leaf interveinal chlorosis and reductions in biomass (Hebbern et al., 2009; Schmidt et al., 2016). The recorded *F*_v_*/F*_m_ values of control plants were maintained close to the theoretical optimum of 0.83 throughout the experimental period (**Table 1**). However, *F*_v_*/F*_m_ decreased drastically in response to Mn deficiency, reaching values of 0.7, 0.6, and 0.5 in the mild, moderate, and strong Mn deficiency treatments, respectively. The measured *F*_v_*/F*_m_ values corresponded to Mn leaf concentrations of around 9 μg Mn g^-1^ DW and around 6.5 μg Mn g^-1^ DW under mild and moderate Mn deficiency, respectively, whereas strong Mn deficiency correlated with values of 3.8 and 5.3 μg Mn g^-1^ DW for Antonia and Vanessa, respectively (**Table 1**). Furthermore, the multi-element ICP-MS analysis showed sufficient concentrations of all other essential plant nutrients as evaluated on the basis of established threshold concentrations (data not shown).

After having established that chlorophyll fluorescence is a reliable tool to monitor the degree of Mn deficiency, we used this tool to cultivate plants with similar levels of Mn deficiency in both genotypes. These plants were used for a more detailed characterization of the photosynthetic apparatus in response to Mn deficiency by evaluating the corresponding OJIP fluorescence transients. Furthermore, NPQ responses under various light intensities were analyzed together with the PSII subunit composition at three stages of Mn deficiency. The results are described in the following sections.

### Mn Deficiency Leads to Appearance of a K Peak and D Dip in the OJIP Fluorescence Transients

**FIGURE 1:**
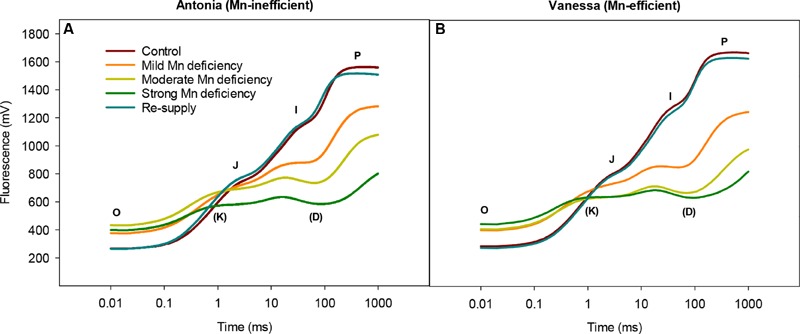
**Manganese deficiency leads to marked changes in the function of the photosynthetic apparatus.** Fluorescence induction kinetics in the youngest fully emerged leaf of the Mn-inefficient barley genotype Antonia **(A)** and the Mn-efficient genotype Vanessa **(B)** growing at Mn-replete (control) or under mild, moderate, or strong Mn deficiency, followed by re-supply of Mn. Averaged transients from each treatment are illustrated (*n* = 12-24). See **Table 1** for the corresponding maximum quantum yield of photosystem II (PSII; *F*_v_*/F*_m_) and the resulting leaf Mn concentrations in each treatment.

The measurements of fluorescence induction kinetics showed that Mn deficiency caused an increase in the minimal fluorescence, *F*_0_ and a concomitant decrease in the maximal fluorescence, *F*_m_ (**Figure 1**). This indicates a defect in the electron transfer within PSII, leading to declining *F*_v_*/F*_m_ values (**Table 1**).

Also the general shape of the OJIP fluorescence transient curves showed distinct alterations in response to Mn deficiency (**Figure 1**). With intensified Mn deficiency, a pronounced K step developed between 0.2 and 0.4 ms. A similar development of the K step was observed after heat-treatment of plants and was attributed to inhibition of the OEC of PSII (Toth et al., 2007). The K step became increasingly prominent with increasing severity of Mn deficiency (**Figure 1**) implying more damage to the OEC under these conditions. While the I step plateau remained unchanged, a D dip appearing between 75 and 90 ms after the I step became deeper as Mn deficiency became stronger (**Figure 1**).

No clear differences in any of the kinetic steps could be observed between the two genotypes except that the fluorescence induction transients declined faster for the Mn-efficient cv. Vanessa than for the Mn-inefficient cv. Antonia (**Figure 1**). Notably, a normal OJIP transient curve could be restored by re-supplying Mn to plants experiencing strong Mn deficiency (**Figure 1**), demonstrating that fully functional OEC’s were present in the newly developed leaves at about 8–10 days after Mn addition to the nutrient solution.

### Mn Deficiency Lowers the Ability of the Plant to Induce Photoprotection Mechanisms

**FIGURE 2:**
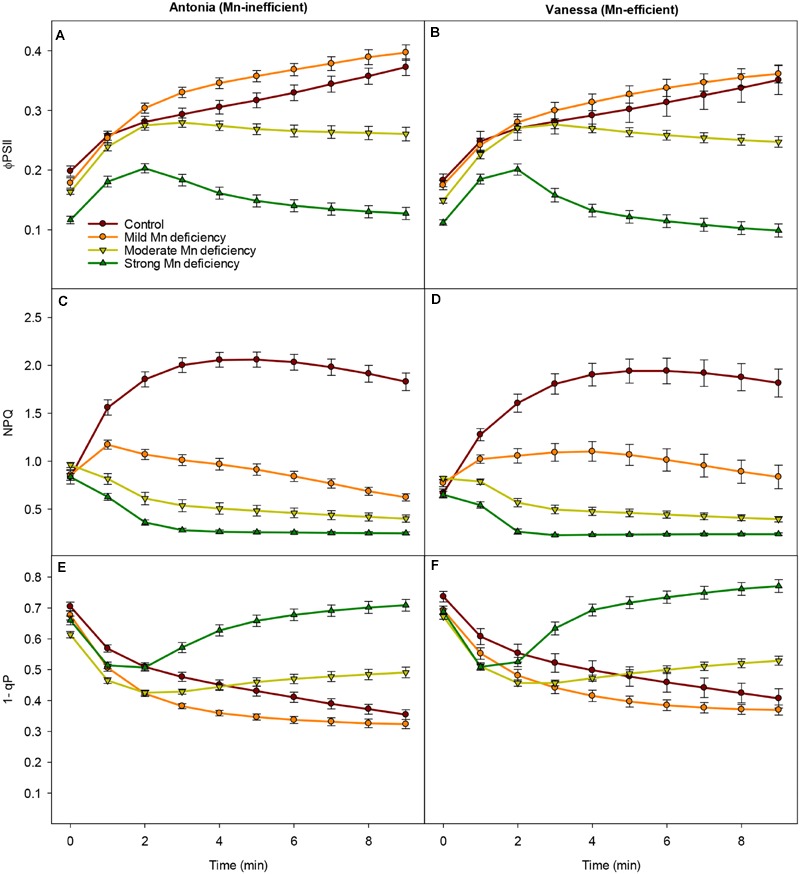
**Photosystem functions are inhibited under growth light conditions.** Effective quantum yield (Φ_PSII_), non-photochemical quenching (NPQ) and PSII excitation pressure (1-q_p_) measured on the youngest fully emerged leaf of Mn-replete (control) and Mn-deficient (mild, moderate, or strong) plants of the Mn-inefficient barley genotype Antonia **(A,C,E)** and the Mn-efficient genotype Vanessa **(B,D,F)** exposed to constant growth light (300 μmol photons m^-2^ s^-1^) conditions. Values are means ± SE (*n* = 13–30).

The impact of Mn deficiency on photoprotection mechanisms was examined by conducting a saturating pulse (SP) quenching analysis under growth light conditions, i.e., 300 PAR (μmol photons m^-2^ s^-1^). A marked reduction in the Φ_PSII_ was observed, constituting up to 70% under strong Mn deficiency (**Figures 2A,B**). This supports the drastic decrease in PSII efficiency observed in the OJIP transients (**Figure 1**). Plants with moderate and strong Mn deficiency were not able to increase the Φ_PSII_ as shown by the decreasing response as a function of time (**Figures 2A,B**).

Also NPQ was strongly affected by Mn, leading to declining NPQ values in Mn deficient plans, while in Mn replete (control) plants NPQ initially increased with the onset of illumination (**Figures 2C,D**). In general for both genotypes, the more Mn deficient the plants became, the lower NPQ values were recorded (**Figures 2C,D**). Strong Mn deficiency resulted in NPQ values reaching only 10% of control levels, indicating that Mn deficient plants were unable to dissipate excess absorbed energy. Notable, the steady-state levels of NPQ were maintained longer under mild Mn deficiency in leaves of Vanessa compared to Antonia although under moderate and strong Mn deficiency the genotypes were equally affected as the plants were unable to induce NPQ under these stress conditions (**Figures 2C,D**).

After 2 min of illumination with growth light there was a clear increase in PSII excitation pressure (1-qP) for plants exposed to moderate and strong Mn deficiency (**Figures 2E,F**), indicating a strongly reduced plastoquinone (PQ) pool. By contrast, the PSII excitation pressure for plants grown under mild Mn deficiency or control conditions continued to decrease showing the ability of these plants to better handle the absorbed light in a controlled way and with a greater PSII efficiency (**Figures 2E,F**). In general, the excitation pressure of PSII was slightly higher for the Mn-efficient genotype Vanessa compared to the Mn-inefficient genotype Antonia across all treatments (**Figures 2E,F**), suggesting that the ability to reduce the PQ pool was less affected in the Mn-efficiency genotype Vanessa.

**FIGURE 3:**
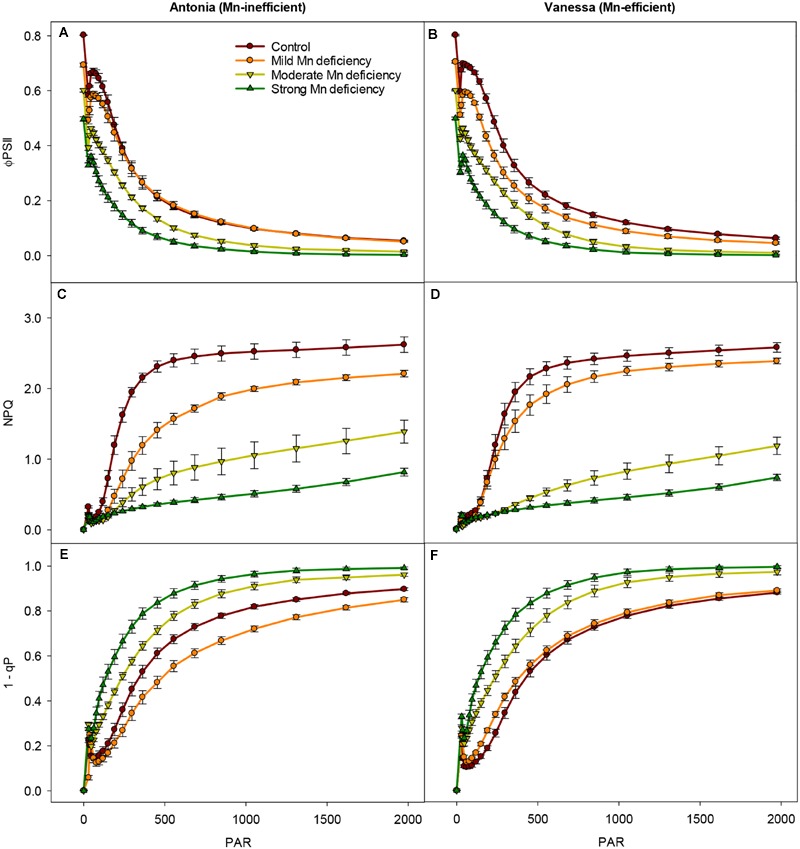
**Changes in photosynthetic capacity during Mn deficiency.** Light-response curves for the effective quantum yield (Φ_PSII_), NPQ, and PSII excitation pressure (1-q_p_) measured on the youngest fully emerged leaves of Mn-replete (control) and Mn-deficient (mild, moderate, or strong) plants of the Mn-inefficient barley genotype Antonia **(A,C,E)** and the Mn-efficient genotype Vanessa **(B,D,F)** exposed to light intensities increasing from 0 to 1976 μmol photons m^-2^ s^-1^ (PAR). Values are means ± SE (*n* = 6).

Photosynthetic performance under various light intensities was measured (**Figure 3**), in order to investigate how increasing light intensities in combination with Mn deficiency influence the photosystem functionality, including regulation of NPQ mechanisms and the PSII excitation pressure (1-qP). The recorded light response curves revealed that for both genotypes the Φ_PSII_ declined faster and more pronouncedly with increasing Mn deficiency than was the case for control plants (**Figures 3A,B**). When comparing the light induced responses for the two genotypes in more detail, only minor differences were detected. However, the mild Mn deficiency and control treatments resulted in similar quantum yield-response curves for the Mn-inefficient genotype Antonia whereas these treatments differed for the Mn-efficient genotype Vanessa where the mild Mn deficient plants revealed a generally lower Φ_PSII_ than the control plants at all light intensities.

Interestingly, the ability to induce NPQ, and thereby the ability to regulate light-harvesting, was in both genotypes markedly decreased at moderate and strong Mn deficiency, the reduction reaching 90% at strong Mn deficiency (**Figures 3C,D**). The induction of NPQ in control and mild Mn deficiency plants showed a saturating response in relation to increasing PAR levels, whereas moderate and strong Mn deficiency resulted in a linear response implying a defect in establishment of NPQ under these conditions. In particular, more drastic reductions in NPQ induction levels were observed for the Mn-inefficient Antonia under all light intensities compared to Vanessa under mild Mn deficiency, indicating a reduced electron flow through PSII (**Figures 3C,D**). More specifically, Vanessa maintained around 20% higher NPQ values compared to Antonia under mild Mn deficiency, indicating an increased resistance of Vanessa plants toward photoinhibition under these conditions. No genotypic differences were observed between plants grown at control conditions or under strong Mn deficiencies, but under moderate Mn deficiency Vanessa was more affected (up to 25% reduction in NPQ) compared to Antonia (**Figures 3C,D**).

The impact of high light intensities on photosynthetic performance was further elucidated by calculation of the PSII excitation pressure (1-qP; **Figures 3E,F**). A significant increase (up to 50%) in 1-qP was observed for both genotypes at moderate and strong Mn deficiency. However, at mild Mn deficiency the excitation pressure for Antonia was lower than that for both Vanessa and for control plants (**Figures 3E,F**).

### Mn Deficiency Restricts the Restoration of PSII Functionality after Photoinhibitory Light Treatment

**FIGURE 4:**
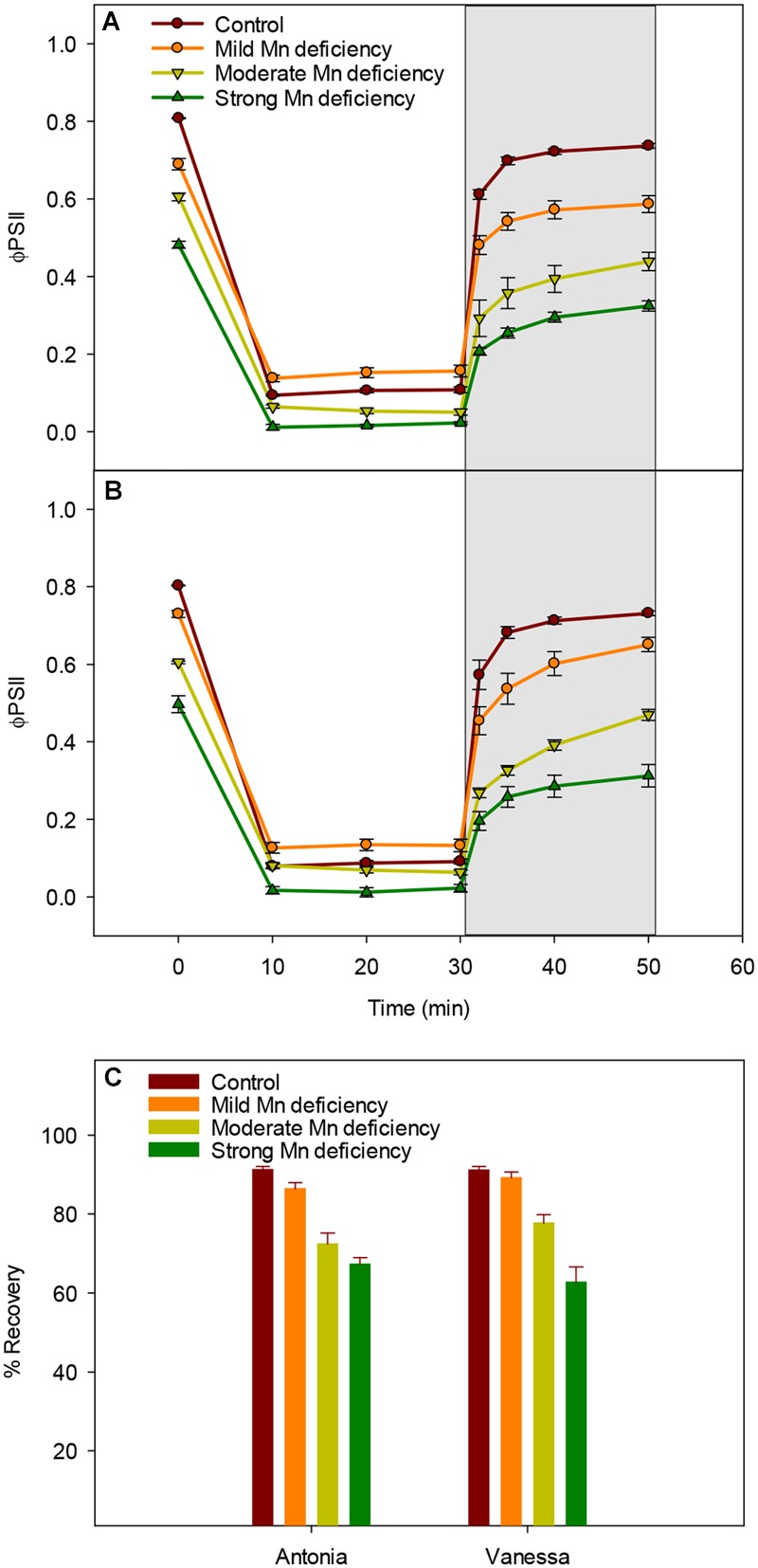
**Manganese deficiency decreases recovery from photoinhibition. (A)** and **(B)** Dark/light induction curves and recovery periods for the effective quantum yield (Φ_PSII_) measured on the youngest fully emerged leaf of Mn-replete (control) and Mn-deficient (mild, moderate, or strong) plants of the Mn-inefficient barley genotype Antonia **(A)** and the Mn-efficient genotype Vanessa **(B)** exposed to 1000 μmol photons m^-2^ s^-1^ (PAR) for 30 min followed by 20 min recovery in darkness (marked in gray). **(C)** The percentage recovery after photoinhibition for each treatment was calculated based on the initial Φ_PSII_ values at time zero. Values are means ± SE (*n* = 6).

High light intensities clearly challenged the photosynthetic apparatus and increased the degree of photoinhibition under Mn deficiency (**Figure 3**). Hence, we investigated the ability of plants to restore PSII functionality when exposed to high light induced photoinhibition at increasing levels of Mn deficiency (**Figure 4**). As expected, the Φ_PSII_ was drastically reduced in leaves exposed to 1000 μmol photons m^-2^ s^-1^ for 10 min (**Figures 4A,B**). The reduction relative to control plants was most pronounced in strongly Mn deficient plants (**Figures 4A,B**). Subsequently, control plants were able to regain up to 90% of the initial PSII quantum yield measured prior to the light treatment, whereas plants with strong Mn deficiency only reached to approximately 60% of the initial value (**Figure 4C**). Interestingly, for Vanessa plants exposed to mild and moderate Mn deficiency the kinetics of recovery was slightly faster which resulted in 5–10% higher Φ_PSII_ values compared to Antonia (**Figure 4C**). Mn deficiency (mild and moderate levels) thus resulted in less overall damage to the photosynthetic apparatus in the Mn-efficient genotype Vanessa than in the Mn-inefficient genotype Antonia, suggesting differences in coping with light stress between the two genotypes under Mn deficiency.

### The OEC Protein Composition Is Affected by Mn Deficiency

**FIGURE 5:**
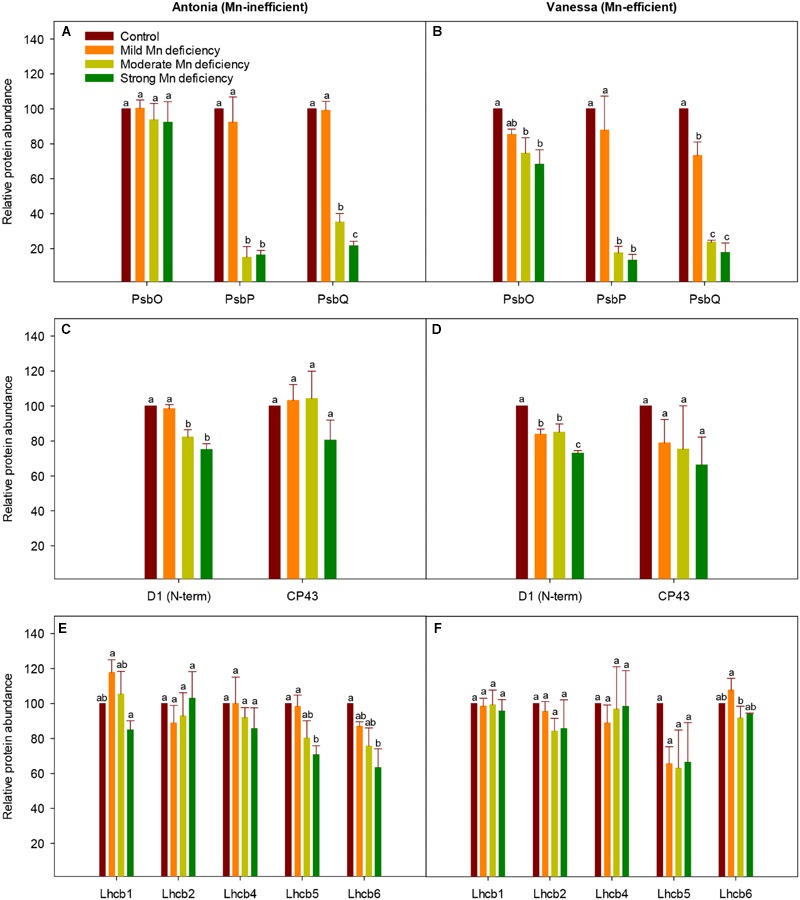
**Changes in PSII subunit composition during Mn deficiency.** Western blot analysis of PSII proteins in replete (control) and Mn-deficient (mild, moderate, strong) leaves of the Mn-inefficient genotype Antonia **(A,C,E)** and the Mn-efficient genotype Vanessa **(B,D,F)** using antibodies specific for OEC subunit proteins PsbO, PsbP, and PsbQ **(A,B)**, PSII core subunits D1 and CP43 **(C,D)** and PSII minor antenna proteins Lhcb4 (CP29), Lhcb5 (CP26), and Lhcb6 (CP24) together with the major antenna proteins Lhcb1 and Lhcb2 **(E,F)**. Values are means ± SE (*n* = 3-4). Bars with the same letter are not significantly different (*P* ≥ 0.05).

The PSII subunit composition was investigated by western blot analysis in the two genotypes under increasing Mn deficiency (**Figure 5**). The abundance of the extrinsic proteins PsbP and PsbQ was dramatically reduced, whereas only minor alterations in PsbO abundance were observed (**Figures 5A,B**). More specifically, the accumulation of PsbP and PsbQ was reduced to 16 and 13% for PsbP and to 22 and 18% for PsbQ of control levels, for the Mn-inefficient genotype Antonia and the Mn-efficient genotype Vanessa, respectively. The strong response in PsbP and PsbQ abundance toward Mn deficiency correlates with leaf Mn concentrations (**Table 1**). However, the abundance of PsbP and PsbQ seem highly variable at incipient Mn deficiency rendering it difficult to demonstrate clear differences between genotypes (**Supplementary Figure S1**). Even so, a notable observation was that the Mn-efficient genotype Vanessa appears to react and adjust more sensitively in response to increasing Mn deficiency compared to the Mn-inefficient genotype Antonia (**Figures 5A,B**). To further investigate the effects of Mn deficiency on PSII subunit composition, the levels of representative PSII core proteins and LHC subunits were investigated. Interestingly, only minor, but significant, reductions in abundance of the PSII core protein D1 were observed in both genotypes as a response to increasing Mn deficiency (**Figures 5C,D**). Likewise, the abundance of the minor antenna complexes found closest to the PSII core complexes (Lhcb4-6) (**Figures 5E,F**) seem to adjust to Mn deficiency in accordance with the D1 core protein of PSII (**Figures 5C–F**). By contrast, the levels of the proteins Lhcb1 and Lchb2, subunits of the major antenna complexes, LHCII, remain unaffected, even in the case of strong Mn deficiency (**Figures 5E,F**). These results support the visual observation that the plants stayed green without any visual leaf symptoms no matter the degree of Mn deficiency throughout the experimental period. Finally, changes in selected PSI subunits (PsaA, PsaD, and PsaF) were recorded with about 20% decrease in the abundance going from control to strong Mn deficiency for both genotypes (data not shown). It is important to note that no genotypic differences in the composition of PSII core or peripheral antenna complex proteins could be observed.

## Discussion

The aim of this study was to analyze and compare changes in photochemistry and PSII subunit composition induced by increasing levels of Mn deficiency in two barley genotypes differing in Mn efficiency. Plants with mild, moderate, or strong Mn deficiency were established and perturbations in the photosynthetic apparatus were assessed by fluorescence measurements. The chlorophyll fluorescence transients displayed the characteristic O-J-I-P kinetic steps, which indicated the individual rate limiting steps of the photosynthetic electron transport chain (Stirbet and Govindjee, 2011). Mn deficiency induced an increase in *F*_0_ indicating a higher level of photoinhibition due to detachment of LHCII complexes (Husted et al., 2009). Mn deficiency cause destabilization of PSII and the amount of PSII is decreased whereas the amount of LHCII complexes remain relatively unchanged (**Figure 5**) causing an imbalance between PSII cores and peripheral antennas. Detachment of LHCII as a consequence of Mn deficiency further supports the fact, that Mn deficient leaves remain green until the deficiency becomes severe, which is why Mn deficiency appears and is commonly referred to as a latent disorder under field conditions. However, the possibility that detachment of LHCII is part of a regulatory adaptation process to balance excitation pressure on PSII cannot be excluded. Further interpretation of the OJIP transients (**Figure 1**) revealed, that Mn deficiency resulted in the development of two additional kinetic steps, designated as the K step and the D dip, respectively (**Figure 1**). This is in accordance with the observations in our previous work (Husted et al., 2009; Schmidt et al., 2013). The precise physiological basis of the K step is still not fully understood, but it is widely accepted that it reflects damage to the OEC (Strasser, 1997; Bukhov et al., 2004) and the K step has previously been observed in plants subjected to heat stress (Srivastava et al., 1997; Strasser, 1997). In the present study, we show that the development of the K step is consistent with the fact that Mn deficiency resulted in major disturbances in the protein composition of OEC as observed by the marked decrease in the abundance of PsbP and PsbQ (**Figures 5C,D**). In addition, the observed D dip under Mn deficiency has been suggested to reflect a partially or fully dismantled Mn cluster (**Figure 1**) (Pospisil and Dau, 2000). Hence, the stability and functioning of the photosynthetic machinery is highly dependent on an intact OEC including all the extrinsic proteins. Together these results suggest that PsbP (and PsbQ) may serve a coordinating function in the assembly/disassembly of the Mn cluster during PSII biogenesis or under PSII repair (De Las Rivas et al., 2007; Järvi et al., 2013; Cao et al., 2015).

Non-photochemical quenching declined significantly in Mn deficient plants of both genotypes (**Figures 2** and **3**), indicating that the donor side of PSII was under increased stress along with impairment and/or lack of upregulation of processes in photoprotection. A major part of NPQ consists of the xanthophyll cycle (Niyogi et al., 2001) and impairment of PSII induced by Mn deficiency prevent build up and maintenance of the translumenal pH gradient crucial for initiating the xanthophyll cycle (Müller et al., 2001). However, NPQ measurements in two different light regimes, viz. growth light intensities (300 μmol photons m^-2^ s^-1^ PAR) and light intensities increasing from 0 to 1920 μmol photons m^-2^ s^-1^ PAR, indicated that the impact of mild Mn deficiency for the Mn-efficient genotype Vanessa resulted in less damage to the photosynthetic apparatus than was the case in the Mn-inefficient genotype Antonia (**Figures 2** and **3**). This is consistent with the fact that the ability to perform state transitions under conditions with mild Mn deficiency only decreased significantly in the Mn-inefficient genotype Antonia, and not in the Mn-efficient genotype Vanessa (Husted et al., 2009). However, under the mild Mn deficiency, the electron transport and therefore lumen acidification is only marginally affected and hence NPQ is mainly a result of the xanthophyll cycle. Furthermore, under mild Mn deficiency, PSII is still being repaired to some extent as verified by **Figures 5A–D** showing that the abundance of D1, PsbO, PsbP, and PsbQ is still about 80–90% of control under these conditions, and hence, photoinhibition is low.

Sustained reduction in NPQ will eventually lead to photoinhibition and the kinetics of recovery from photoinhibition was markedly slowed down for plants with Mn deficiency compared to control plants (**Figure 4**). The observed recovery of Φ_PSII_ (**Figures 4A,B**) may suggest relaxation of NPQ, but not necessarily recovery from photoinhibition as this process may require several hours. Moreover, the fact that NPQ in leaves of Mn deficient plants is not regulated to the same degree as in control plants, implies damage to PSII. By leaving the damaged PSII complex in an inactive state, the nearby PSII complexes are protected against damage, as the photoinhibited PSII complexes during repair acts like quenchers of excitation energy (Chow et al., 2002; Matsubara and Chow, 2004). The level of photoinhibition and recovery of PSII is a balance between damage and repair of the PSII holocomplex (Kirchhoff, 2014). Measurements of fluorescence using pulse amplitude modulation (**Figures 2–4**) essentially provide an estimate of the functional steady state pool of PSII but not the actual turnover or differences in repair. Combined with the observation that the Mn-efficient genotype Vanessa was better in coping with mild Mn deficiency as compared to the Mn-inefficient genotype Antonia (**Figures 2–4**), this suggests that a better balance between damage and repair of PSII during continuous Mn deficiency will ensure more functional PSII reaction centers in Mn-efficient genotypes. A significant gradual decrease in the D1 core protein together with a clear decrease in CP43 abundance was observed for the Mn-efficient genotype Vanessa under mild Mn deficiency but not for the Mn-inefficient genotype Antonia (**Figures 5C,D**). These data suggest that Vanessa may control and maintain protein homeostasis by adjusting to mild Mn deficiency conditions better than Antonia. However, under moderate and strong Mn deficiency both genotypes showed severe reductions in PSII core and OEC proteins (**Figures 5A–D**) and genotypic differences disappeared suggesting loss of ability to adjust protein abundance in both genotypes. In this context it was recently shown that the Mn-efficient genotype Vanessa had significantly more Mn bound per unit of PSII under control and mild Mn deficiency conditions than the inefficient genotype Antonia, despite having lower or similar total leaf Mn concentrations (Schmidt et al., 2015). Likewise, as demonstrated in the present study it was found that under more severe Mn deficiency, the differences between the two genotypes disappeared (Schmidt et al., 2015). This points to a better internal utilization of Mn in the Mn-efficient genotype Vanessa under control and mild Mn deficiency, that tends to load and/or preserve more Mn in PSII supercomplexes. It further supports the hypothesis, that the Mn-inefficient genotype has a higher requirement for Mn in photosynthesis. The subtle differences in fluorescence between the two genotypes at moderate and strong Mn deficiency (**Figures 2–4**) may reflect differences in one or more of the steps in the PSII repair cycle, especially concerning the recycling and reassembly of the water oxidizing Mn-cluster.

## Conclusion

We conclude that Mn deficiency reduces photosynthetic performance owing to a damaged and dysfunctional OEC which impairs the transfer of electrons through the reaction centers of PSII. Consequently, the recovery from high light-induced photoinhibition is severely slowed down in Mn deficient plants. Mn deficiency furthermore negatively affects short-term regulatory mechanisms, such as NPQ. The Mn-efficient barley genotype Vanessa is able to maintain a higher and more responsive NPQ than the Mn-inefficient genotype Antonia when plants are exposed to mild Mn deficiency. However, under strong Mn deficiency there are no differences between the two genotypes, indicating a chronic breakdown in the ability to regulate light-harvesting and efficiently repair PSII.

## Author Contributions

SS, MP, KK, BN-B, and PP designed and carried out all experimental work. SS, PJ, and PP drafted the manuscript. JS and SH contributed to conceiving the study, data interpretation and discussion and helped to revise the manuscript. All authors have read and approved the final manuscript.

## Conflict of Interest Statement

The authors declare that the research was conducted in the absence of any commercial or financial relationships that could be construed as a potential conflict of interest.

## Supplementary Material

FIGURE S1 **Example of western blot analysis used as data input for **Figure 5**.** Western blot analysis of thylakoids prepared from replete (control) and Mn-deficient (mild, moderate, strong) plants. Shown are three replications using antibodies specific for CF1-APTase included as a loading control and the OEC subunit PsbP. Lane 1–4, 9–12, and 17–20 are replicates of the Mn-inefficient genotype Antonia and lane 5–8, 13–16, and 21–24 represent replicates of the Mn-efficient genotype Vanessa presented in the in the order control, mild, moderate, and strong Mn deficiency.
