# Supplementary figures and images for: Photosystem II Functionality in Barley Responds Dynamically to Changes in Leaf Manganese Status
Source: Front Plant Sci. 2016 Nov 25;7:1772. doi: 10.3389/fpls.2016.01772 (PMC5122584; doi:10.3389/fpls.2016.01772)

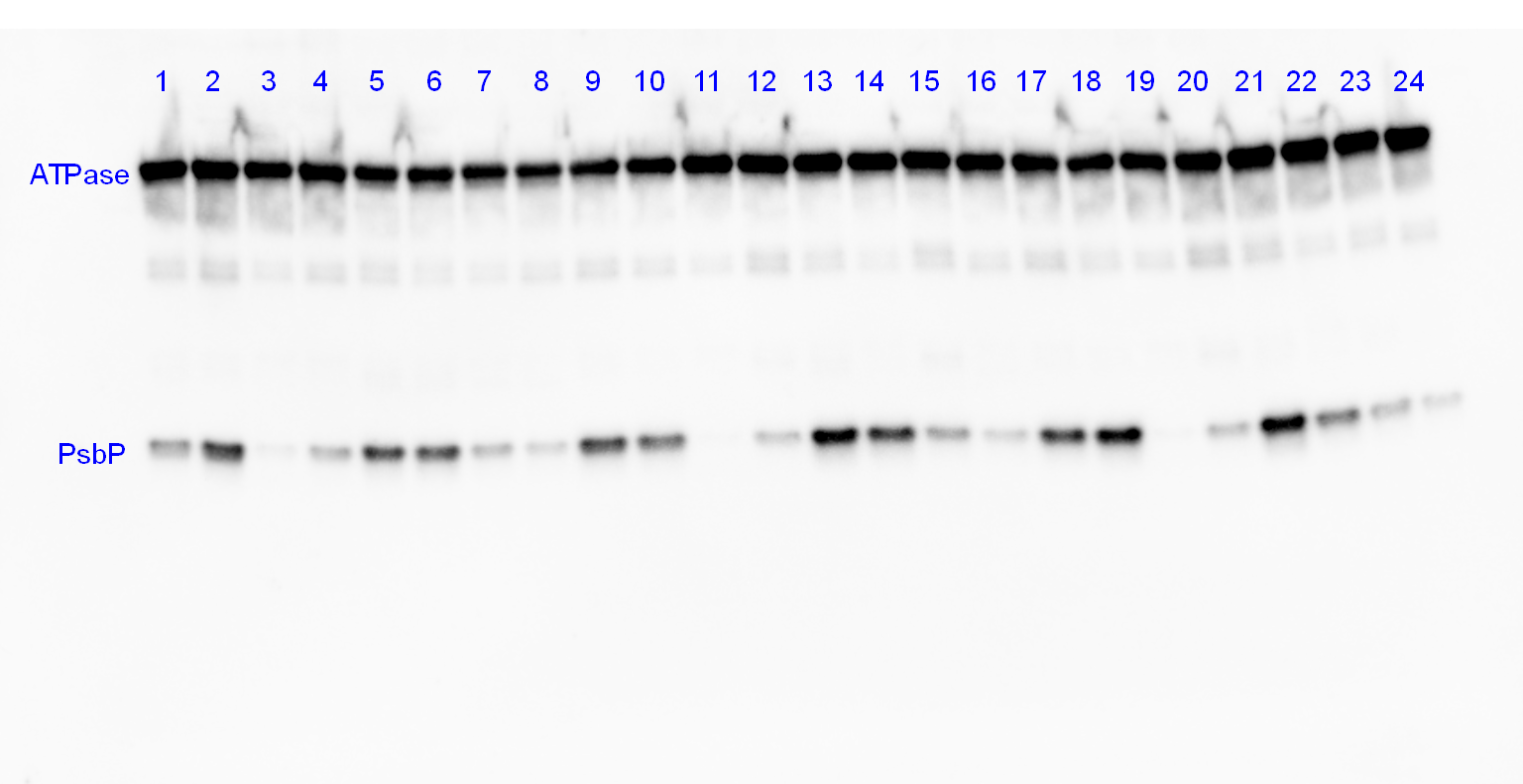

Supplement: FIGURE S1 — Example of western blot analysis used as data input for Figure 5. Western blot analysis of thylakoids prepared from replete (control) and Mn-deficient (mild, moderate, strong) plants. Shown are three replications using antibodies specific for CF1-APTase included as a loading control and the OEC subunit PsbP. Lane 1–4, 9–12, and 17–20 are replicates of the Mn-inefficient genotype Antonia and lane 5–8, 13–16, and 21–24 represent replicates of the Mn-efficient genotype Vanessa presented in the in the order control, mild, moderate, and strong Mn deficiency. [file Image_1.TIF]
